# Perilla Seed Oil and Protein: Composition, Health Benefits, and Potential Applications in Functional Foods

**DOI:** 10.3390/molecules29225258

**Published:** 2024-11-07

**Authors:** Lijun Guan, Ling Zhu, Xindi Zhang, Yaxi Han, Kunlun Wang, Nina Ji, Xinmiao Yao, Ye Zhou, Bo Li, Qing Chen, Jing Fan, Dixin Sha, Shuwen Lu

**Affiliations:** 1 Institute of Food Processing Research, Heilongjiang Province Academy of Agricultural Sciences, Harbin 150086, China; 2 Heilongjiang Province Key Laboratory of Food Processing, Harbin 150086, China; 3 Institute of Soya Research, Heilongjiang Province Academy of Agricultural Sciences, Harbin 150086, China

**Keywords:** perilla seed oil, perilla seed protein, composition, health benefits, application

## Abstract

Perilla (*Perilla frutescens*) seeds are emerging as a valuable resource for functional foods and medicines owing to their rich oil and protein content with diverse nutritional and health benefits. Perilla seed oil (PSO) possesses a high level of a-linolenic acid (ALA), a favorable ratio of unsaturated to saturated fatty acids, and other active ingredients such as tocopherols and phytosterols, which contribute to its antioxidant, anti-inflammatory, and cardiovascular protective effects. The balanced amino acid ratio and good functional properties of perilla seed protein make it suitable for a variety of food applications. The chemical composition, health benefits, and potential applications of PSO as well as the structural characterization, functional properties, modification methods, bioactivities, and application scenarios of perilla seed protein are comprehensively presented in this paper. Furthermore, the challenges as well as future prospects and research focus of PSO and perilla seed protein are discussed. The growing interest in plant-based diets and functional foods has made PSO and perilla seed protein promising ingredients for the development of novel foods and health products. The purpose of this paper is to highlight implications for future research and development utilizing these two untapped resources to improve human health and nutrition.

## 1. Introduction

**Figure 1:**
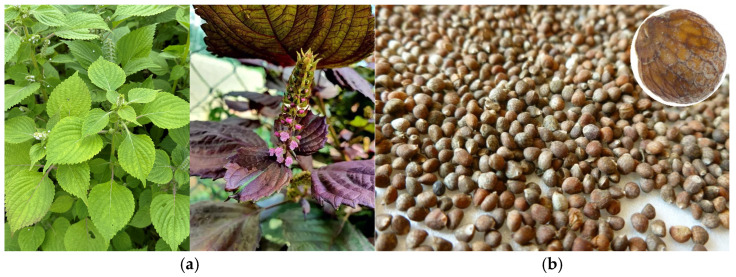
The plant (**a**) and seed (**b**) of perilla.

Perilla (*Perilla frutescens* L. Britton), a medicine and food homologous plant, is a kind of aromatic plant belonging to the Labiatae family [1]. Perilla has green or purple leaves, produces small self-pollinating flowers and brown fruits, and contains black to white as well as varied shades of gray or brown seeds (Figure 1) [1,2]. The leaves and seeds of perilla have long been utilized for their unique flavor and aromatic qualities, adding a distinctive flavor to culinary dishes. In addition to its culinary applications, perilla is valued for its medicinal properties. The dried whole plant of perilla exhibits effects such as hemostasis, pain relief, cough suppression, detoxification, anti-inflammatory action, and laxative properties [1,3]. Perilla is widely distributed in China, Korea, Japan, and other Asian regions [4,5]. In 2019, perilla seeds were harvested from an area of 429.1 hectares in Japan, yielding a total production of 137.9 tons [6], while before 2000, the annual production of perilla seeds in Korea was 36,800 tons, and by 2015, the annual output of perilla seeds could reach 46,000 tons [7]. Due to its escalating economic significance, perilla is now also being cultivated by Western countries, including the United States, Russia, and various European nations [8]. Perilla is growing momentum for large-scale applications in a variety of fields.

Perilla seeds are an ideal raw material for edible oil, with an oil content ranging as high as 35% to 45% [9]. PSO contains high levels of the essential omega(ω)-3 polyunsaturated fatty acids (PUFAs): ALA (54–64%) [10]. The consumption of ALA-rich oils is effective in promoting human growth and development; improving diabetes, cardiovascular, and cerebrovascular diseases; and lowering blood lipids and cholesterol [11]. In addition to PUFAs, PSO is a good source of biologically active compounds like tocopherols, phytosterols, and flavonoids [12]. These active ingredients can be directly absorbed by the human body, making PSO a health-promoting functional plant oil for human consumption [13].

A plant-based high-protein diet may reduce the risk of type II diabetes associated with the long-term high intake of animal protein. As a result, plant proteins are receiving increasing focus [14]. High levels (20–30%) of protein are also found in perilla seeds [15]. Perilla seeds are pressed for oil to form perilla seed meal (PSM) by-products, which are commonly employed as animal feed or discarded outright. Nevertheless, PSM contains 35–45% protein content, and this treatment has resulted in the loss of the functional components of perilla protein [16]. Therefore, it is imperative to conduct research on perilla seed protein to enhance the utilization of perilla seeds. Perilla seed protein holds immense potential for a wide range of applications owing to its high protein efficiency and excellent functional properties [17]. Studies have shown that perilla seed protein could be used to encapsulate and deliver unstable actives such as β-carotene and curcumin, as well as being developed as packaging materials for meat products [9,18,19].

In light of the growing interest in plant-based diets and functional foods, perilla seed oil and perilla seed protein are emerging as valuable resources with diverse nutritional and health benefits. The chemical composition, health benefits, and application scenarios of PSO, as well as the composition, structure, functional properties, modification techniques, biological activities, and potential applications of perilla seed protein in the food sector are described in this paper. The aim of this paper is to provide a basis for the further development of perilla seed resources as an emerging functional food to improve human health.

## 2. Methodology

The literature search was conducted using multiple scientific databases, including Web of Science, PubMed, Scopus, ScienceDirect, and Google Scholar, covering publications from 1990 to 2024. The primary keywords used were “perilla seed oil”, “perilla seed protein”, “*Perilla frutescens*”, “composition”, “health benefits”, and “applications”. Peer-reviewed articles in English focusing on the composition, properties, and applications of perilla seed oil and protein were included in the criteria. Non-English papers, conference abstracts, and studies lacking proper experimental validation were excluded.

## 3. Perilla Seed Oil

### 3.1. Chemical Composition of Perilla Seed Oil

**Figure 2:**
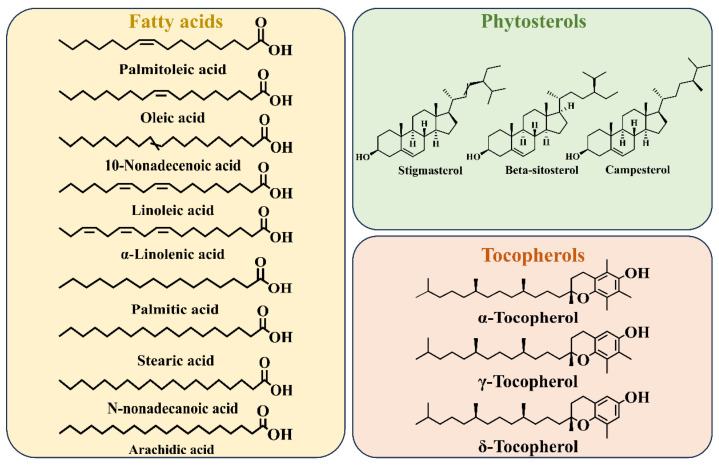
Chemical structure of fatty acids, phytosterols, and tocopherols in PSO.

The nutritional worth of vegetable oil depends on the composition of the fatty acids [20]. Research has shown that PSO is composed of a wide range of fatty acids (Figure 2), with unsaturated fatty acids (UFAs) and saturated fatty acids (SFAs) accounting for 91.70% and 8.34%, respectively [21]. The UFA/SFA ratio of PSO is well above two, suggesting that PSO has a hypolipidemic function [22]. The monounsaturated fatty acids (MUFAs) were determined to be palmitoleic acid (C16:1) (0.13–0.21%), oleic acid (C18:1) (12.50–13.52%), and 10-Nonadecenoic acid (C19:1) (0.07%); the PUFAs were linoleic acid (C18:2) (13.02–13.75%) and α-linolenic acid (C18:3) (63.32–64.41%); and the SFAs were palmitic acid (C16:0) (7.02–7.23%), heptadecanoic acid (C17:0) (0.08%), stearic acid (C18:0) (1.41–1.45%), N-nonadecanoic acid (C19:0) (0.09%), and arachidic acid (C20:0) (0.18%) [23].

Among the edible vegetable oils, PSO possesses more than 60% ALA content in comparison to siritch oil (49.03%), peony seed oil (40.83%), and herbaceous peony seed oil (30.84%) [22]. As a member of the ω-3 PUFAs family, ALA could be converted into eicosapentaenoic acid (EPA) and docosahexaenoic acid (DHA) by desaturases and elongases, promoting the development of the brain and retina [24,25]. Linoleic acid (LA) belongs to ω-6 PUFAs. Research has demonstrated that edible oils with an ω-6/ω-3 PUFA ratio of less than four were more healthful and could lower the incidence of cancer and cardiovascular events [11]. PSO included the lowest ω-6/ω-3 PUFA ratio compared to several other edible vegetable oils (flax seed oil and chia seed oil) [26]. The high UFA/SFA ratio and low ω-6/ω-3 PUFA ratio suggest that PSO is a prospective healthy edible vegetable oil.

Phytosterols and tocopherols are essential ingredients in vegetable oils.

The inclusion of phytosterols in the human diet is recommended due to their cholesterol-lowering, anti-inflammatory, anti-bacterial, and anti-tumor abilities [27]. According to the determination of Li et al. [28], PSO contained stigmasterol, β-sitosterol, and campesterol, with the contents of 105.25, 3186.12, and 186.58 mg/kg oil, respectively. Pan et al. [27] similarly found β-sitosterol to be the most abundant phytosterol in PSO. Vitamin E (tocopherols) can serve as a free radical scavenger in membranes and lipoproteins and is a native oil-soluble antioxidant [29]. PSO has been identified in several studies to contain three kinds of tocopherols, including α-tocopherol, γ-tocopherol, and δ-tocopherol [28,30]. The biological activity of α-tocopherol is the highest among the eight vitamin E congeners [31]. In addition, γ-tocopherol is considered to have better antioxidant activity than α-tocopherol, while δ-tocopherol exhibits only 1% bioavailability of α-tocopherol [22,29]. In PSO, γ-tocopherol (661.79–730.83 mg/kg oil) has the highest content of tocopherols, followed by δ-tocopherol (72.44–80.59 mg/kg oil) and α-tocopherol (5.36–19.77 mg/kg oil) [27]. In addition to the above components, PSO has been identified to contain phenolic [30], pyrazine [32], aldehyde [20], and flavonoid compounds [33]. The chemical composition of PSO has been found to be significantly influenced by the extraction methods employed. A comparative analysis of the major chemical components of PSO obtained through different extraction methods is presented in Table 1.

**Table 1:** Chemical composition of PSO by different extraction methods.

Extraction Method	Condition	Yield/ Efficiency	Chemical Composition	References
Atmospheric pressing extraction	40 °C	33.17 g/100 g	Fatty acid: C16:0 (5.69 ± 0.00%), C18:0 (2.43 ± 0.06%), C18:1 (16.00 ± 0.00%), C18:2 (13.20 ± 0.00%), and C18:3 (63.33 ± 0.00%)	[34]
Vacuum pressing extraction	40 °C, 2.67 kPa oxygen-free environment, and 30 min	33.15 g/100 g	Fatty acid: C16:0 (5.69 ± 0.01%), C18:0 (2.41 ± 0.10%), C18:1 (16.03 ± 0.02%), C18:2 (13.17 ± 0.03%), and C18:3 (63.30 ± 0.02%)	[34]
Solvent extraction	80% (*v*/*v*) ethanol solution, liquid/solid ratio of 10 mL/g, 85 °C, 4 h, and 3 repeats	39.48%	12 fatty acids, the main fatty acids were ALA (63.32%), C18:1 (12.50%), and C18:2 (13.75%)	[23]
	Methyl acetate, liquid/solid ratio of 10 mL/g, and 4 h	27.76 ± 0.23%	10 fatty acids, the main fatty acids were ALA (66.51 ± 0.02%), C18:2 (17.77 ± 0.02%), and C16:0 (10.15 ± 0.04%)	[20]
	Petroleum ether	—	Stearic acid (9.6%), palmitic acid (16.1%), linoleic acid (11.9%), oleic acid (18.3%), and ALA (44.1%); total phenolics (28.7 mg/100 g); total flavonoids (12.3 mg/100 g)	[35]
Ultrasound-assisted extraction	400 W of ultrasonic power, 41.26 °C, 17.11 min, and liquid/solid ratio of 7.02:1	36.27%	SFAs (6.99%), MUFAs (16.76%), and PUFAs (76.25%); linolenic (63.93%), linoleic (12.32%), and oleic acid (16.65%); the content of stigmasterol, β-sitosterol, and campesterol was 105.25, 3186.12, and 186.58 mg/kg, respectively; the content of α-tocopherol, γ-tocopherol, and δ-tocopherol was 33.52, 453.88, and 10.85 mg/kg, respectively	[28]
Tea saponin-induced ultrasonic-assisted extraction	Ultrasonic power of 90 W, 16 min, tea saponin dose of 0.3%, 40 °C, 80% (*v*/*v*) ethanol, and liquid–material ratio of 10:1 mL/g	39.10 ± 2.13%	12 fatty acids, the main fatty acids were ALA (64.41%), C18:1 (13.52%), and C18:2 (13.75%)	[23]
Aqueous enzymatic extraction	Solid/water ratio of 1:4, pH 6, 55 °C, enzyme loading of 2%, and hydrolysis time of 4.5 h	Maximum oil recovery was 88.52%	ALA (63.2%)	[36]
Ultrasound-assisted aqueous enzymatic extraction	250 W of ultrasonic power, 30 min, and 50 °C	Cellulase (81.74%); Alcalase 2.4 L (61.84%); Protex 6 L (62.31%); Protex 7 L (61.25%)	Fatty acid: C16:0, C18:0, C18:1, C18:2, C18:3, and C20:0	[37]
	Liquid/solid ratio of 4.4:1, hydrolysis time of 2.66 h, hydrolysis temperature of 50.87 °C, and ultrasound treatment time of 24.74 min	31.34%	Fatty acid: C16:0 (4.43 ± 0.05%), C18:0 (1.87 ± 0.01%), C18:1 (20.39 ± 0.15%), C18:2 (9.12 ± 0.08%), and C18:3 (64.05 ± 0.20%); 450.88 mg/kg of total tocopherols; 615.25 mg GAE/kg of total phenolics	[38]
Microwave-assisted extraction	385 W, 30 min, and liquid–material ratio of 10 mL/g	32.66 ± 0.14%	10 fatty acids, the main fatty acids were ALA (66.59 ± 0.02%), C18:2 (17.83 ± 0.01%), and C16:0 (10.33 ± 0.03%)	[20]
Stepwise microwave hydrodistillation and extraction	70% moisture content, 10 min and 700 W of first-stage microwave-mediated process, 15 mL/g of liquid-material ratio, and 15 min and 385 W of second-stage microwave-mediated process	36.49% ± 0.07%	8 fatty acids, ALA (69.90 ± 0.03%), linoleic acid (16.37 ± 0.02%), and palmitic acid (9.23 ± 0.01%)	[20]
Supercritical CO_2_ fluid extraction	45.61 °C, 26.72 MPa of pressure, and the perilla seed ratio of 0.36	7.43 ± 0.15 g/60 g sample	49.14 ± 1.38% for ALA; total polyphenol content of 0.51 mg GAE/g oil	[39]
	Pressure 33.98 MPa, 42 °C, and CO_2_ flow rate 29.25 L/h.	37.53 ± 0.37%	Stearic acid (3.4%), palmitic acid (7.5%), linoleic acid (5.9%), oleic acid (4.5%), and ALA (78.7%); total phenolics (130.4 mg/100 g); total flavonoids (35.3 mg/100 g)	[35]
Compressed fluid extraction	Compressed CO_2_	31.80%	17 fatty acids, the major fatty acid was linolenic (56.19–62.80%), linoleic acid (14.34–15.19%), and oleic acid (12.02–14.94%); squalene (0.87–6.12 mg/100 g); α-tocopherol (6.60–12.48 mg/100 g); β-sitosterol (56.32–72.61 mg/100 g)	[40]
	Compressed LPG [a mixture of propane (50.3%), n-butane (28.4%), isobutane (13.7%), ethane (4.8%), and other minor constituents (methane, pentane, and isopentane)]	42.29%	17 fatty acids, the major fatty acid was linolenic acid (57.03–57.55%), linoleic acid (14.77–15.19%), and oleic acid (15.04–14.70%); squalene (0.54–6.04 mg/100 g); α-tocopherol (4.65–7.23 mg/100 g); β-sitosterol (12.72–44.88 mg/100 g)	[40]

“—” stands for not given in the literature.

### 3.2. Health Benefits of Perilla Seed Oil

The main health benefits of PSO, such as antioxidant, anticancer, anti-inflammatory, cardiovascular and cerebrovascular protection, liver protection, and anti-diabetic, are summarized in Table 2.

#### 3.2.1. Antioxidant

PSO is considered a natural antioxidant capable of scavenging free radicals and mitigating oxidative stress. The in vitro antioxidant activities of PSO were estimated using the 2,2-diphenyl-1-picrylhydrazyl (DPPH) and 2,2′-azino-bis (3-ethylbenzothiazoline-6-sulfonic acid) diammonium salt (ABTS) radical scavenging assay and reducing power assay. Upon measurement, the EC50 (the concentration of sample required to reduce the absorbance at the measurement wavelength by 50%) values for the clearance of DPPH and ABTS by PSO were found to be 7.01 mg/mL and 12.75 mg/mL, respectively, and the AC50 (the concentration of the sample required when the absorbance value reached 0.5 at measurement wavelength) for reducing capacity was 4.3 mg/mL [35]. It was shown that the scavenging rate of PSO on DPPH radical was proportional to its concentration [23,38]. The ability of PSO to scavenge DDPH free radicals was associated with polyunsaturated fatty acids, phenolic compounds, carotenoids, and tocopherols [27,38]. Oxidative stress is crucial in cognitive decline [41,42]. The supplementation of PSO could significantly improve oxidative stress in mice with amyloid beta (25–35)-induced cognitive dysfunction (Alzheimer’s disease, AD) by inhibiting lipid peroxidation and excessive nitric oxide production in the brains, livers, and kidneys of mice [43]. The beneficial effects of PSO on oxidative stress in AD mice were superior to those of olive oil and corn oil due to its richness in ALA [44]. The consumption of PSO (7.0 mL, 12 months) in diet could enhance antioxidant capacity and protect against age-associated decline in cognition and intelligence in healthy older adults through increasing blood ALA levels [45]. In addition, a 12-month intake of POPP (1.47 mL of PSO and1.12 g of ponkan powder) could elevate serum brain-derived neurotropic factor and antioxidant potential, potentially improving age-associated cognitive deficits in healthy elderly individuals by increasing erythrocyte ω-3 PUFAs levels [46]. ALA-rich PSO may prevent or treat neurodegenerative diseases by exerting its antioxidant capacity [47].

#### 3.2.2. Anticancer

Cancer is a key worldwide health issue contributing to high incidence and mortality rates [48]. ω-3 PUFAs are considered as potential dietary factors for the chemoprevention of cancer [49]. As early as the 1990s, epidemiological and experimental studies found that adding ω-3 PUFA-rich PSO to diet suppressed the risk of colon and breast cancers [50,51,52]. In the azoxymethane-induced F344 rat model of colonic aberrant crypt foci, dietary PSO (10% and 20%) inhibited the formation of large foci (with a crypt multiplicity of four or more), with a suppression rate of 38–53%. Furthermore, dietary PSO (20%, 12 weeks) inhibited the activity of cyclooxygenase (COX-1 and COX-2) in intestinal tumors and reduced the level of the colonic mucosal metabolite prostaglandin (PG)E2, leading to a suppression of >69% and >52% in the development of small intestinal and colon tumors in adenomatous polyposis coli (APC) mice, respectively [53]. The mechanism of colonic tumor inhibition by PSO is partly mediated by the modulation of COX activities through ALA. ω-3 PUFAs can also enhance the efficacy and tolerance of chemotherapy, to some extent assisting in the treatment of cancer [54].

#### 3.2.3. Anti-Inflammatory

**Figure 3:**
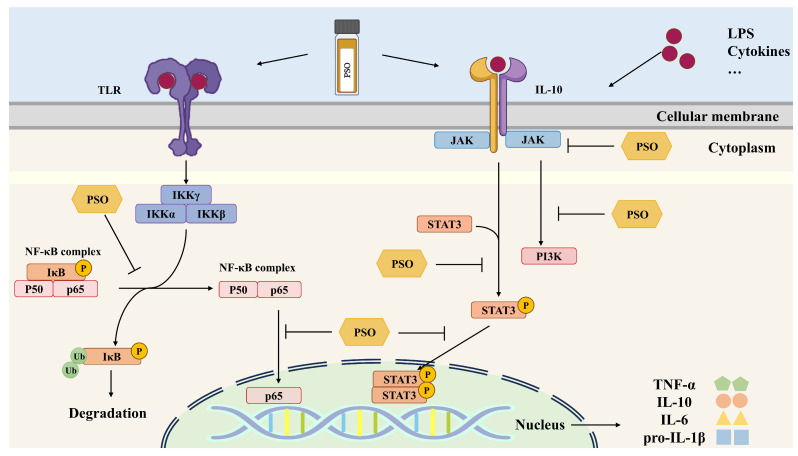
Activation and negative regulation of JAK-STAT and NF-κB signaling pathways after PSO treatment.

Inflammation is the body’s initial defense against infections and injuries caused by physical and chemical trauma, medications, and infective microorganisms, used to remove damaged tissue or intruding pathogens [55,56]. The anti-inflammatory action of PSO at a dose of 8% (*w*/*w*) taken orally has been researched in in vivo assays using high-fat -diet (HFD)-induced colon inflammation mice. PSO effectively reduced the levels of pro-inflammatory cytokines interleukin (IL)-1β and IL-6, increased the expression levels of epithelial integrity barrier markers (tight junction protein Claudin-1 and Zo-1), and attenuated colonic inflammation by inhibiting the NF-κB pathway [57]. In rats with colitis, ALA in PSO exerted an anti-inflammatory action by increasing the tissue levels of EPA and reducing the levels of arachidonic acid [58]. The administration of PSO (50, 100, and 500 mg/kg/d) could effectively attenuate ecological dysregulation, inflammation, and metabolic disorders in the gut of obese rats induced by an HFD [59]. The effect of PSO on attenuating the inflammatory response in obese mice induced by HFD was associated with an ω-3/ω-6 fatty acid ratio of 1:2 [60]. Moreover, the anti-inflammatory activity of PSO also shows great potential in regulating lung function. Dietary PSO probably attenuates allergic asthma inflammation through suppressing the secretion of pro-inflammatory cytokine IL-1β, IL-6, and tumor necrosis factor (TNF)-α in the airways, lungs, and spleen of mice with allergic airway inflammation [61]. The supplementation of PSO inhibited the release of leukotriene B4 (LTB4) from calcium ionophore-stimulated polymorphonuclear neutrophils (PMNs), exerting anti-asthmatic activity in capsaicin-induced asthmatic rats by suppressing the biosynthetic pathway of LTB4 and anti-inflammatory activity [62]. The relevant signaling pathways are shown in Figure 3.

#### 3.2.4. Benefits for Cardiovascular and Cerebrovascular Health

Deaths caused by cardiovascular and cerebrovascular disease are projected to increase to 6.1 million by 2030 [29]. Vegetable oils are considered better than animal fats for cardiovascular and cerebrovascular disease [63]. The effective effects of PSO on cardiovascular and cerebrovascular health have been demonstrated in multiple studies owing to its richness in ALA. The addition of PSO to the diet could ameliorate HFD-induced hyperlipidemia in rats [64]. Research revealed that feeding PSO lowered total blood cholesterol (TC) and low-density lipoprotein (LDL) levels, significantly reduced cerebral hemorrhage and infarct damage in stroke-prone spontaneously hypertensive rats, and prolonged the survival period of rats from 58 to 68.5 days [65]. Jo et al. [66] found that the change in the plasma triglyceride (TG) concentrations of subjects (20–35 years old) who had rice porridge containing emulsified PSO (RPEPO) for breakfast (333 kcal/meal) for 30 days was significantly lower compared to those who had rice porridge for breakfast (333 kcal/meal). A clinical trial conducted by Wei et al. [67] involving 36 participants (18–75 years old) with hyperlipidemia explored the effects of the administration of PSO capsules (4 grain/time, twice/day) on serum lipid and C-reactive protein (CRP) levels. The experimental data showed a significant reduction in the lipid and CRP levels in patients after 56 days of PSO treatment, suggesting that PSO has the ability to protect endothelial cells and reduce the likelihood of atherosclerosis. Compared to middle cerebral artery occlusion (MCAO) rats fed other plant oils (canola or sesame oils) and trans fats, the long-term feeding of PSO could lower blood lipids and fat accumulation, and significantly reduce brain damage and physical functional impairments in MCAO rats [63]. Hence, it is recommended that PSO can be the top option for modern dining tables that consume a lot of fats and oils.

#### 3.2.5. Other Beneficial Effects

In addition to the above health benefits, PSO has been reported to possess the ability to protect the liver, treat diabetes, improve bone health, act as a laxative, and prevent the photoaging of the skin. The study showed that PSO suppressed hepatic steatosis in HFD-induced obese mice through inhibiting lipogenesis and lipolysis in liver tissue [68]. The ameliorative effect of PSO on HFD-induced non-alcoholic fatty liver disease and intestinal dysbiosis was similar to that of fish oil, but the effectiveness was slightly weaker than fish oil [69]. In a mouse model of type 2 diabetes mellitus (T2DM) induced by a high-fat and high-sugar diet in combination with a single low-dose streptozotocin, PSO intervention (1.84 g/kg) decreased fasting blood glucose, TG, and TC levels, increased insulin levels, activated glucose transporter 4 (Glut4) expression, and restored the diversity of the intestinal microbiota. PSO regulated the gut microbiota and alleviated insulin resistance in T2DM mice via the PI3K/AKT signaling pathway [70]. ω-3 PUFAs are beneficial for bone mineral density (BMD). Matsuzaki et al. [71] indicated that the daily intake of PSO (7.0 mL, 12 months) significantly increased BMD levels, serum ALA levels, and serum biological antioxidant potential to diacron reactive oxygen metabolite ratios, and decreased tartrate-resistant acid phosphatase 5b levels in adults (mean age 54.2 ± 6.4 years) when compared to daily olive oil intake (7.0 mL, 12 months). Long-term PSO consumption might improve bone health by inhibiting bone resorption and increasing ALA levels. Another study found that PSO could serve as a laxative to stimulate intestinal excretion by increasing the volume and water content of feces in constipated Swiss albino rats without causing diarrhea [72]. In addition, the topical application of PSO inhibited wrinkle formation and water loss in the dorsal skin of UV-exposed hairless mice, preventing skin cell damage and photoaging through the modulation of skin morphology, tissue pathology, and antioxidant capacity [73].

**Table 2:** Multiple health benefits of PSO.

Health Benefits	In Vitro/In Vivo Model	Doses/Duration	Effects	References
Antioxidant	DPPH	5–50 μL/mL	Scavenging rate over 80% (50 μL/mL)	[23]
	DPPH, ABTS, and reducing power	—	DPPH EC50 = 7.01 mg/mL; ABTS EC50 = 12.75 mg/mL; reducing power AC50 = 4.3 mg/mL	[35]
	DPPH	0–10 mg/mL	IC50 was 3.99 (ultrasound-assisted aqueous enzymatic extraction oil, 4.80 (solvent extraction oil), and 7.19 mg/mL (cold pressing extraction oil), respectively	[38]
	Amyloid beta (25–35)-induced AD mice	ALA separated from PSO, 100 mg/kg/day, 14 days	Lipid peroxidation and nitric oxide overproduction in the brain, liver, and kidney of mice ↓	[43]
	Healthy older Japanese (64–84 years old)	7 mL, 12 months	Serum biological antioxidant potential (BAP) level ↑	[45]
	Healthy elderly volunteers	1.47 mL PO and 1.12 g *Anredera cordifolia* leaf powder, 12 months	Red blood cell plasma membrane ALA and EPA levels, and serum BAP levels ↑; serum TG, glucose, and *N*-(epsilon)-carboxymethyl-lysine levels ↓	[10]
	Healthy elderly individuals (60–85 years old)	1.47 mL of PO and 1.12 g ponkan powder	Serum brain-derived neurotrophic factor and BAP, and red blood cell ω-3 PUFAs ↑	[46]
Anticancer	Azoxymethane-induced colonic aberrant crypt foci F344 rat	10% and 20%, 12 weeks	The number of foci with ≥4 crypts/focus, development of small intestinal and colon tumors, and cyclooxygenase (COX)-1 and COX-2 activities ↓	[53]
	Colon carcinogenesis rat	12%, 0–35 weeks	Ornithine decarboxylase activity and colon tumor development ↓	[50]
	F344 rat	12%, 35 weeks	Incidence of colon cancer reduced from 67% to 32%	[54]
Anti-inflammatory	HFD-induced colon inflammation mice	8%	TNF-α, IL-1β, and IL-6 levels ↓, and tight junction protein (Claudin-1 and Zo-1) expression levels ↑; the initiation of NF-κB signaling pathway	[57]
	Dextran sulfate sodium-induced ulcerative colitis rat	—	The tissue level of EPA ↑; arachidonic acid ↓	[58]
	HFD-fed rats	50, 100, and 500 mg/kg/day, 12 weeks	TNF-α, IL-1β, MDA, and lipopolysaccharide (LPS) levels ↓; reducing intestinal barrier dysfunction, systemic inflammation, and hyperlipidemia in HFD-fed rats	[59]
	Female C57BL/6J mice	HFD plus PSO, 8 weeks	Abdominal adipose and uterine adipose tissue weights, JAK2, STAT3, and SOCS3 mRNA expression, and p-NF-κB and IL-6 levels ↓	[60]
	Ovalbumin (OVA)-sensitized and -challenged allergic asthmatic inflammation mice	50% PSO and 50% corn oil, 35 days	The levels of TNF-α, IL-1β, and IL-10 decreased by 47.3%, 62.9%, and 62.7%, respectively	[61]
Benefits for cardiovascular and cerebrovascular health	Asthma rats	1.0, 2.0, and 4.0 g/kg	The inhibition rates of LTB4 release by PMNs were 11.1%, 24.4% and 57.9%, respectively	[62]
	High cholesterol diet-fed Apolipoprotein (Apo) E knock-out (KO) mice	1.25% cholesterol and 10% PSO, 10 weeks	Inducible nitric oxide synthase activity decreased by 20.8%; plasma cholesterol ↓; fibrinolysis and endothelial nitric oxide synthase activity ↑	[74]
	Young adults (20–35 years old)	RPEPO, 333 kcal/meal, 30 days	Plasma triglyceride ↓	[66]
	Adults [18–75 years old, TC) ≥ 6.22 mmol/L or TG ≥ 2.26 mmol/L or low-density lipoprotein-cholesterol (LDL-C) ≥ 4.14 mmol/L]	PSO capsules, 4 grain/time, twice/day, 56 days	Serum TG, TC, and LDL-C; TNF-α; plasminogen activator inhibitor-1; and highly sensitive C-creative protein levels ↓	[67]
	Cerebral ischaemic rats	10%	Blood TG, TC, and LDL levels, and cerebral infarction ↓	[63]
liver protection	HFD-induced obesity C57BL/6N mice	10 mL/kg, 16 weeks	Liver weight, liver fat accumulation, and hepatic steatosis ↓	[68]
	HFD-induced nonalcoholic fatty liver disease rats	5.5%, 16 weeks	Minor reversal of severe hepatic steatosis and significant reduction in the relative abundance of Gram-positive bacteria in the intestine; TNF-α, IL-1β, and IL-6 levels ↓; serum alanine aminotransferase ↑	[69]
Anti-diabetic	Diabetic KKAy mice	0.67, 1.33, and 2.00g/kg, 12 weeks	Improvement of hepatocellular macrovesicular steatosis and adipocyte hypertrophy; serum TG and intestine *Blautia* ↓	[75]
	T2DM mice	1.84 g/kg	The levels of fasting blood glucose, TG, TC, glucose, and glucose-6-phosphate dehydrogenase, lipid droplets accumulation, and weight loss ↓; the levels of insulin, ALT, and AST ↑; the regulation of PI3K/AKT signaling pathway	[70]
Improve bone health	Adults (mean age 54.2 ± 6.4 years)	7.0 mL/day, 12 months	Tartrate-resistant acid phosphatase 5b ↓; serum biological antioxidant potential/diacron reactive oxygen metabolite ratio and ALA level ↑; suppressing bone resorption	[71]
Laxative	Swiss albino rats	5 and 10 mL/kg	Stimulating intestinal emptying; the weight and water content of feces ↑	[72]
Anti-skin aging	UV-induced photoaging of normal human dermal fibroblast (NHDF) cells and SKH-1 hairless mice	0.625%, 1.25%, and 2.5% for NHDF cells; 50 and 100 μL for mice	Significantly ameliorated the UV-induced reduction in cell viability and SOD activity, production of ROS, and arrest of arrest; the inhibition of wrinkle formation in dorsal skin, transepidermal water loss, and the increment of melanin index in mice	[73]

“—” stands for not given in the literature; “↑” stands for improvement; “↓” stands for decrease.

### 3.3. Application of Perilla Seed Oil

Traditionally, PSO has been utilized as an edible oil for the direct cooking of foods to balance the ω-6/ω-3 PUFA ratio in the diet, and as a functional ingredient in food products [2]. Studies showed that the addition of PSO to potato blueberry-flavored yogurt (PBY) enhanced the amounts of amino acids, total viable bacteria, and flavor substances in the yogurt and improved the sensory quality [76,77]. The enzymatic transesterification process could be utilized to synthesize PSO into trans-fat-free structured lipid-rich ω-3 PUFAs, with potential applications in baked goods and dairy products [12]. Replacing animal fats with a mixed emulsion of PSO and rapeseed oil, which have good emulsification stability, lower cooking losses, and texture properties similar to animal fats, could reduce the fat content and enhance the health value of meat emulsion products [78]. The complete replacement of animal fats by pre-emulsified PSO-canola oil reduced fat, calorie, and ω-6/ω-3 PUFA ratio in chicken sausage, thereby being used in the production of low-fat chicken sausages [79]. PSO was also demonstrated to be effective in stabilizing the gel of meat products. Compared with soybean oil, the larger fat globules of PSO could be more uniformly dispersed in the gel structure, improving the denseness and homogeneity of the gel structure, and thus enhancing the thermal stability of surimi gels [80]. Furthermore, PSO has a beneficial effect on animal feed. Feeding diets supplemented with PSO could reduce blood lipid levels, improve fatty acid composition, and increase the overall chicken meat acceptability in yellow-feathered chickens [81]. Guo et al. [82] found that the addition of PSO to the feed had better effects on improving muscle mass, total antioxidant capacity, and immunity; promoting lipid metabolism; and maintaining the health of the liver and intestinal tract of Chinese giant salamanders compared with the addition of soybean oil and fish oil. The addition of PSO to the culture medium significantly promoted the growth of *Cunninghamella elegans* (TISTR 3370). The supplementation of PSO resulted in a higher total lipid production compared to soybean oil and rice bran oil supplementation [83]. The biological activities of PSO, such as antioxidant, anticancer, and anticarcinogenic, suggest that it also has a good prospect for application in the pharmaceutical field, which needs to be further investigated.

## 4. Perilla Seed Protein

### 4.1. Composition and Structure of Perilla Seed Protein

Perilla seeds are full of protein (20–30%), similar to mung beans, peas, and almonds [14,15,84,85]. Defatted perilla seed (perilla seed meal) has a protein content of 35–40%, which can be used as a high-protein food [86]. Research has revealed that perilla protein extracted from perilla seed meal exhibits elevated nutritional value and superior protein efficiency, which can be utilized as a novel alternative source of animal protein [87]. Presently, the protein obtained from perilla seed meal is commonly referred to as perilla protein isolate (PPI). According to the report, albumin (11%) and globulin (84%) are the two major protein components in perilla seed meal [88].

Sodium dodecyl sulfate–polyacrylamide gel electrophoresis (SDS-PAGE) is one of the most commonly employed methods for determining the molecular weight of protein and has been used for PPI by many scholars. Based on the literature, the molecular weight distribution of PPI is relatively broad, ranging from ~1 to 340 kDa [15,88,89,90]. PPI is rich in both the acidic and basic types of subunits [91]. Three main intermediary subunits of perilla globulin have the evaluated molecular weights of 54, 57, and 59 kDa, respectively. The structure of perilla globulin is composed of an acidic subunit and a basic subunit linked by disulfide bonds to form an intermediary subunit, while six pairs of intermediary subunits associate to form a 340 kDa globulin molecule [88].

As far as the composition of amino acids in perilla proteins is concerned, there are subtle differences between different studies (Table 3). For instance, Zhao et al. [87] identified 17 amino acids in perilla protein, including tyrosine (Tyr), valine (Val), phenylalanine (Phe), isoleucine (Ile), leucine (Leu), and lysine (Lys), which are the six essential amino acids. Hydrophobic and acidic amino acids are the major constituents of PPI. Hu et al. [92] discovered that perilla protein contains all the essential amino acids for adults except tryptophan (Trp). Glycine (GlY), Phe, and Leu are the dominant amino acids in perilla seed protein. The perilla seed protein was determined to have an acidic character due to high levels of aspartic acid (10.40 ± 0.08%) and glutamic acid (Glu) (27.03 ± 0.07%) [93]. Perilla protein has a balanced content of essential amino acids, with adequate proportions of all the essential amino acids except Lys [88]. Therefore, perilla protein has great potential for development.

### 4.2. Functional Properties of Perilla Seed Protein

The functional characteristics of proteins such as solubility, emulsification, water/oil holding capacity, and foaming affect the textural and sensorial properties of food and are necessary in the manufacture of food products [94]. The functional properties of perilla proteins are summarized in Table 3.

#### 4.2.1. Solubility

Solubility is the most essential property of protein which significantly influences other functional properties such as gelation, emulsification, and foaming capacity [95]. Typically, proteins with higher solubility possess better gelation, emulsification, and foaming ability [94]. Protein solubility is defined as the protein concentration in the solvent when protein–protein and protein–solvent interactions reach thermodynamic equilibrium [96]. The solubility of perilla protein is pH-dependent and shows a “U-shaped” trend, with the greatest solubility at pH 10 and the least solubility at pH 4–5, which suggested that the isoelectric point of perilla protein is pH = 4–5 [87,97,98]. At the isoelectric point, the protein carries zero net charge and has no electrostatic repulsion force. The hydrophobic interactions between proteins lead to protein aggregation and precipitation, thus reducing the solubility [99,100]. When deviating from the isoelectric point, the surface charge density of protein molecules increases. Due to electrostatic repulsion, the solubility of proteins will increase [84]. Soya and pea proteins have similar u-shaped solubility profiles with minimum solubility values between pH 4 and 5 [101].

#### 4.2.2. Emulsifying Ability

Proteins, owing to their amphiphilic properties, can function as emulsifiers by resisting the emulsification, coaling, settling, and flocculation of droplets [95]. Emulsification behavior indicates the protein molecule’s ability to adsorb at the oil–water interface, which is of vital importance for the utilization of protein in the food industry [102]. Homogenized perilla protein has been proven to be an effective stabilizer for high internal phase Pickering emulsions (HIPPEs) [103]. Emulsion activity (EA) and stability (ES) are two major indices used to evaluate emulsification performance [104]. EA quantifies the adsorption capacity of a protein at an interface and is determined by the maximum emulsified oil volume per unit weight of protein, while ES assesses the performance of the formed adsorption layer, defined as anti-emulsification within a particular duration [100,105]. Perilla protein has good emulsifying properties. According to the literature, the EA of perilla protein ranged from 8.07 to 3393 m^2^/g. It was shown that the EA and EI of perilla protein exhibit pH dependence. As the pH of the protein dispersion increased from 3.0 to 11.0, the EA and EI of perilla protein initially decreased, followed by an increase, reaching a maximum value at pH 11.0 (EA: 3393 m^2^/g, EI: 172.23 min) [87]. The above phenomenon is consistent with the findings of Park and Yoon [106]. There has been a similar report on the pH-dependent changes in the EA and ES of *Amygdalus pedunculata* Pall seed proteins [107]. In addition to pH, the emulsifying properties of perilla protein are also influenced by heat treatment. Appropriate heat treatment (70 °C) facilitates the unfolding of the perilla protein structure, enhancing its emulsifying performance. During over-heating treatment (90 °C), the thermal denaturation and unfolding of perilla protein lead to the expansion of nonpolar and sulfhydryl groups, which increases protein–protein interactions and thereby causes droplet aggregation [97].

#### 4.2.3. Water/Oil Holding Capacity

Water-holding capacity (WHC) and oil-holding capacity (OHC) represent the ability of proteins to hold water and oil volumes [108]. It was reported that the WHC of protein in perilla seeds defatted by hot-pressing, cold-pressing, and petroleum ether solvent extraction methods were found to be different, measuring 1.89, 2.17, and 1.83 g/g, respectively, while the difference in OHC was not significant [87]. Zhao et al. [89] determined the WHC of perilla protein to be 0.99 g/g, which was considerably less than that of soybean isolate protein (4.36 g/g) and black bean protein (2.9 g/g). In terms of OHC, perilla protein was determined to be 1.76 g/g, which is similar to cowpea protein concentrate (1.75 g/g), but lower than soybean protein (6.09 g/g) and Bambara groundnut protein (6.0 g/g) [91,109]. Based on the above, perilla protein exhibited relatively weak water-holding and oil-holding capacities. Therefore, modification techniques can be employed to enhance the WHC and OHC of perilla protein.

#### 4.2.4. Foaming Properties

Protein is considered to be a pretty good foaming agent [110]. Because of the existence of free energy, foams are thermodynamically unstable at the gas–liquid interface [111]. Protein solutions are able to disperse and adsorb to the gas–liquid interface, with hydrophilic groups bound to the liquid phase and hydrophobic groups orienting towards the gas phase, thus decreasing the superficial tension as well as causing the formation of a viscous film surrounding bubbles [112]. The capacity of proteins to generate stable foam is crucial for producing good-quality foods [113]. The foaming capacity (FC) and foaming stability (FS) of perilla proteins were below 10% as determined by Zhao et al. [114]. In general, proteins with high solubility are more prone to diffuse to the air–water interface, which promotes foaming [107]. Zhao et al. [87] found that pH value significantly affects the FC and FS of perilla protein. The nearer the pH is to the isoelectric point of perilla protein, the poorer the FC and FS are. This finding is attributed to the fact that perilla protein possesses the lowest solubility at the isoelectric point. In the vicinity of the isoelectric point, the FC is less than 40% and FS is less than 10%. From a data perspective, the foaming ability of perilla protein is weaker than that of Lupin protein (350%) and almond kernel protein (>100%) [14,115].

**Table 3:** The content, amino acid composition, molecular weight, and functional properties of perilla seed protein.

Source	Content	Amino Acid Composition	Molecular Weight (kDa)	Solubility	EA	ES	WHC (g/g)	OHC (g/g)	Foaming	References
Perilla seed	—	—	~ 10; 20; 35; 55	48.19%	8.07 m^2^/g	50 min	—	—	FC: 41%; FS: 50–60%	[15]
	White perilla: 24.18%; brown perilla: 25.38%	—	—	—	—	—	—	—	—	[116]
	—	—	Ranging from 10 to 60	65.09% (750 W ultrasonic-treated)	666.33 m^2^/g	42.64 min (750 W ultrasonic-treated)	0.99	2.03	FC: 50%; FS: 30.5% (750 W ultrasonic-treated)	[89]
	25.69%	—	—	55.70%	60.11	—	3.54	1.74	FC: 62.87%	[91]
	138.6 mg/g	Asp; Thr; Ser; Glu; Gly; Ala; Val; Ile; Leu; Tyr; Phe; Lys; Arg	10–100; 10–20	—	—	—	—	—	—	[117]
	23.7%	Asp; Thr; Ser; Glu; Gly; Ala; Val; Cystine (Cys); Met; Ile; Leu; Tyr; Phe; His; Lys; Arg; Pro;	11.23; 6.58; 3.83; 2.44; 1.40; 0.42; 0.07	—	—	—	—	—	—	[90]
	—	—	Ranging from 10 to 55	22.54%	1266.65			2.35	FC: 5.63 g/g	[114]
Perilla seed meal	39.5% (*w/w*); albumin ratio: 11%; globulin ratio: 84%	Asx; Thr; Ser; Glx; Pro; Gly; Ala; Cys; Val; Met; Ile; Leu; Tyr; Phe; Lys; His; Arg; Trp	340; 59; 57; 54	—	—	—	—	—	—	[88]
	85.18%	Asp; Glu; Pro; Gly; Ala; Val; Met; Ile; Leu; Tyr; Phe; His; Lys; Arg	1–27	91.69% (alcalase hydrolyzed, pH 7.0); 63.332% (neutrase hydrolyzed, pH 9.0); 94.47% (trypsin hydrolyzed, pH 7.0); 94.57% (papain hydrolyzed, pH 9.0); 93.15 (pepsin hydrolyzed, pH 9.0)	—	—	0.53 (alcalase hydrolyzed); 0.27 (neutrase hydrolyzed); 2.27 (trypsin hydrolyzed); 0.60 (papain hydrolyzed); 0.93 (pepsin hydrolyzed)	3.20 (alcalase hydrolyzed); 2.93 (neutrase hydrolyzed); 5.60 (trypsin hydrolyzed); 5.60 (papain hydrolyzed); 4.13 (pepsin hydrolyzed)	—	[17]
	—	Asp; Glu; Ser; His; Gly; Tyr; Arg; Ala; Cys; Val; Met; Phe; Ile; Leu; Lys; Pro	—	—	3393.0 m^2^/g (pH 11.0)	172.23 min (pH 11.0)	2.17	—	—	[87]
	23.12 mg/mL	—	—	—	—	—	—	—	—	[86]
	65.17%	—	52; 32; less than 6.5	—	—	—	—	—	—	[118]
	—	Amino acid sequence was identified as Ile-Ser-Pro-Arg-Ile-Leu-Ser-Tyr-Asn-Leu-Arg	>10; 5–10; 3–5; <3 kDa	—	—	—	—	—	—	[119]
	10.77%	—	—	—	—	—	—	—	—	[120]
	—	Asp; Thr; Ser; Glu; Gly; Ala; Val; Met; Ile; Leu; Tyr; Phe; His; Lys; Arg	—	39.21% (pH 10.0)	52.46% (pH 2.0)	44.04% (pH 2.0)	1.23	1.36	FC: 4.80 mL (pH 10.0	[93]

“—” stands for not given in the literature.

### 4.3. Modification of Perilla Protein

The functionality or biological activities of perilla protein in its native form are generally suboptimal, which limits their applications. To date, various techniques involving enzyme, physical, and chemical modifications have been reported to enhance the functional properties of perilla protein.

#### 4.3.1. Enzymatic Modification

Enzyme modification has the advantages of being environmentally friendly, having non-toxic by-products, and consuming low energy, making it widely employed for protein modification [95]. This technology hydrolyzes protein macromolecules into smaller peptide fractions primarily using enzymatic endo- and exo-cleavages, thus strengthening or attenuating some of the functional characteristics of the protein [121]. Typically, the enzymatic degradation of protein exposes new ionizable amino acids and carboxyl groups, increasing the reachability of hydrophilic groups as well as promoting the interaction of hydrophilic amino acids and water-based media, thus increasing solubility [106,122]. The solubility of trypsin hydrolysate and papain hydrolysate of perilla protein was determined to be as high as 94.47 ± 2.77% (pH 7) and 94.57 ± 1.94% (pH 9), respectively. Gastric protease acting on the N-terminus of aromatic amino acids can generate perilla protein hydrolysates with angiotensin I-converting enzyme (ACE) inhibitory activity [17]. Furthermore, enzymatic hydrolysis could, respectively, enhance the emulsifying activity and foaming capacity of perilla protein from 44.04 ± 0.82% to 54.68 ± 2.48% and from 4.80 ± 0.26 mL to 6.10 ± 0.10 mL [93].

#### 4.3.2. Chemical Modification

Chemical modification is the chemical introduction of diverse exogenous groups into the protein’s side chain, thereby altering the structure of proteins and facilitating the functional characteristics of proteins [123]. Currently, the chemical modification of perilla protein is primarily focused on phosphorylation modification. Phosphorylation modification is a technology that introduces phosphate groups into the primary structure of a protein to modulate its function [109]. The incorporation of phosphate groups strengthens the electronegativity and electromagnetic repulsion between the molecules of the proteins, making them more easily distributed into water, thereby improving their functional characteristics [124,125]. Sodium tripolyphosphate (STPP), sodium trimetaphosphate (STMP), and phosphorus oxychloride reagents are normally used for the phosphorylation modification of food proteins [109,126]. Zhao et al. [114] modified PPI using various concentrations of STPP and STMP. The study revealed that phosphate groups combine with PPI via C-O-P bonds. Phosphorylation modification significantly improved the thermal stability, solubility, OHC, FC, and EA of PPI, in which the solubility was increased from 22.54% to 92.87% (6% STPP) and 68.51% (4% STMP), and the OHC was increased from 2.35 g/g to 7.80 g/g (8% STPP) and 7.02 g/g (8% STMP). The enhancement of the above functional properties was attributed to the introduction of phosphorylating reagents increasing the particle size and electronegativity of the PPI, leading to the exposing of hydrophobic and free-SH groups and changes in the secondary structure and tertiary conformation of the PPI. Other chemical modifications such as glycation, acylation, and deamidation are also effective techniques to improve the functional properties of proteins, which can be applied to the study of the chemical modification of perilla proteins in the future.

#### 4.3.3. Physical Modification

Physical modification means the adoption of physical methods like heat treatment, high hydrostatic pressure, electric field, magnetic field, acoustic field, and freezing to alter the higher-order structure gathering patterns, thereby changing the functional characteristics of proteins [127,128]. The physical modification of perilla proteins is mainly carried out by ultrasound. Ultrasound technology is based on sound waves with frequencies higher than the human hearing threshold (>16 kHz), which is an environmentally friendly, efficient, and low-equipment-demanding technology [95,129]. It was shown that ultrasound treatment can decrease particle size, expose hydrophobic groups, increase -SH groups, improve the content of α-helix, and reduce the content of β-turn and β-sheet in PPI [89]. The PPI molecules undergo intense collisions due to the strong microfluidic effects generated by the mechanical vibration and cavitation phenomenon of ultrasound, resulting in the formation of a compact structure [15]. These structural changes improve the solubility, OHC, FC, and EA of PPI. In addition, the complexing of perilla protein with other components can lead to the establishment of complexes with new functional features. The covalent binding of perilla protein with polyphenols is beneficial for improving the solubility, thermal stability, and emulsifying properties of perilla protein [98]. The study conducted by Zhao et al. [130] revealed that the composite nanoparticles (LZPI-CS CNPs) formed by the interaction of perilla protein isolate (LZPI) and chitosan (CS) via hydrogen bonding and electrostatic interactions at pH 3.0 could be employed to stabilize HIPPEs due to the provision of steric hindrance and strong electrostatic repulsion. Continuing research has revealed that chitosan–protocatechuic acid (CSPA) coupling facilitated the formation of three-dimensional network structure in high internal phase emulsions (HIPEs) stabilized by LZPI, resulting in good viscoelasticity and storage stability of HIPEs. CSPA was non-covalently bound to LZPI in the formation of LZPI-CSPA complexes by hydrogen bonding and electrostatic interactions. These complexes can generate dense interfacial membranes, thereby retarding lipid oxidation in HIPEs [19].

### 4.4. Biological Activity

Currently, perilla protein has been primarily confirmed to possess antioxidant, anti-diabetic, antihypertensive, fertility-enhancing, and anti-fatigue bioactivities. An imbalance between free radicals and the antioxidant defense system can lead to oxidative stress. Oxidative stress contributes to the development of cardiovascular disease, cancer, and other chronic diseases [131]. Thus, exogenous antioxidant supplementation plays a vital role in mitigating or reducing oxidative stress damage in the body. Perilla protein and their hydrolysates displayed potential as potent antioxidants [17]. For example, Yang et al. [132] isolated two antioxidant peptides (YL with Tyr-Leu sequence; FY with Phe-Tyr sequence) from perilla protein. At peptide concentrations greater than 0.02 mg/mL, their scavenging activity against 2,2′-azino-bis (3-ethylbenzothiazoline-6-sulfonic acid) diammonium salt (ABTS) radicals approached 100%. These two peptides also demonstrated excellent inhibition of lipid peroxidation in rat liver, and their antioxidant activity is attributed to the presence of Tyr residues. The sequence of perilla peptide with 2,2-diphenyl-1-picrylhydrazyl (DPPH) and ATBS radical scavenging activity as well as reducing ability was identified as Ile-Ser-Pro-Arg-Ile-Leu-Ser-Tyr-Asn-Leu-Arg [119]. In a kidney injury mice model induced by adenine, the gavage of perilla peptides at 0.2, 0.3, and 0.6 g/kg for four weeks significantly reduced the levels of alkaline phosphatase (AKP), serum aspartate aminotransferase (AST), and alanine aminotransferase (ALT), creatinine, blood urea nitrogen, and malondialdehyde (MDA), and increased intracellular superoxide dismutase (SOD) activity, the α-diversity of mice intestinal flora, as well as the levels of interleukin (IL)-β, IL-6, and tumor necrosis factor-alpha (TNF-α). Collectively, perilla peptides played an important role in ameliorating adenine-induced apoptotic injury and oxidative stress in the kidney [133]. Flavorzyme-hydrolyzed perilla protein peptides exhibited significant inhibitory activity against α-amylase and ACE, suggesting their potential as anti-diabetic and antihypertensive agents. The fraction with a molecular weight below 1 kDa demonstrates the strongest inhibitory activity [106]. High arginine-containing (9.81%) perilla peptide restored spermatogenesis in mice through activating endogenous spermatogonial cell proliferation, which can be utilized as a medication or a treatment ingredient in dietary prescriptions to enhance the fertility potential of certain individuals with oligoasthenozoospermia [134]. Furthermore, the treatment of mice with perilla seed peptides (0.4, 0.8, and 1.2 kg^−1^ day^−1^) could effectively increase the level of lactate dehydrogenase, muscle coefficient, and exercise endurance of mice, and reduce the levels of blood lactic acid, blood urea nitrogen, and creatinine in blood in a dose-dependent manner. The anti-fatigue activity of perilla peptide was associated with its abundant content of branched-chain amino acids (18.82 g/100 g) and fatigue-reducing amino acids (Glu, Asp, and Arg) [90].

### 4.5. Application of Perilla Seed Protein

In nano-encapsulation technology, perilla proteins and their derivatives have been extensively employed as carriers for preserving and transporting unstable nutrients (Table 4). Perilla seed protein isolate–pectin nano-complexes (PSPI-PEC) assembled using a ph-driven approach could encapsulate 87.77% of curcumin (Cur) [9]. PSPI-Cur-PEC nanocomposites had good biocompatibility and greater resistance to degradation under heat treatment and ultraviolet exposure. Mayonnaise-like HIPPEs produced from LZPI-CS CNPs could serve as three-dimensional feedstock and β-carotene delivery carriers [130]. Furthermore, encapsulating β-carotene with LZPI-CSPA-stabilized HIPEs could improve its retaining ability under various environmental stresses (natural light exposure, ultraviolet irradiation, and heat treatment) [19]. Perilla protein complexed with red algae can be prepared as a composite film with good mechanical properties for packaging pork sausages [18]. Microcapsules prepared by rose essential oil with PPI–sodium alginate (NaAlg) complexes as shell materials could extend the shelf life of ground beef by up to 6 days (4 °C) [135]. It is evident that perilla protein can be used as packaging material for preserving meat products. In addition, perilla protein could form strong bonds with Pb(II) through Vander Waals interactions, serving as a green capturing agent for treating heavy metal-containing wastewater [136].

**Table 4:** Perilla protein nanosystems for delivery and stabilization of biologically active substances.

Embedded Substance	Delivery Vehicles	Processes	Key Findings	References
β-carotene	LZPI-CSPA	β-carotene (0.1 wt%) was dispersed in PSO to form an oil phase, oil phase (80 wt%) mixed with LPZI (2.0 wt%)-CSPA (0.2–2.3 wt%) at high speed, 11,600 rpm for 2 min	Ultraviolet (UV) irradiation (32 h), natural light irradiation (30 d), and heat treatment (45 °C): retention rate increased from 0.33, 0.17, and 0.43 to 0.54, 0.47, and 0.54, respectively	[19]
	Conjugates of perilla seed meal protein (PSMP) with gallic acid (GA), protocatechuic acid (PCA), caffeic acid (CA), apigenin (API), and luteolin (LU), respectively	Oil phase (5 wt%, medium-chain triglyceride containing 0.1% (*w*/*v*) of β-carotene), aqueous phase (95 wt%, PSMP- polyphenol), 13,600 rmp for 5 min, ultrasonic homogenizer for secondary homogenization (600 W, 20 min)	Stability of UV irradiation and heat treatment ↑	[98]
	LZPI-CS CNPs	β-carotene (0.1 wt%) was dispersed in rapeseed oil to form an oil phase, oil phase (80 wt%) mixed with LPZI (4.0 wt%)-CS (0.1–0.6 wt%) at high speed, 12,000 rpm for 2 min	Natural light irradiation (30 d): retention rate increased from 0.18 to 0.53; stability of UV irradiation and heat treatment ↑	[130]
Curcumin (Cur)	PSPI-PEC nano-complexes	The PSPI/Cur mass ratios were set as 80:1, 40:1, 20:1, 10:1, and 5:1, respectively, PSPI/PEC mass ratio was 2:1), pH 4.0, 8000× *g* for 20 min	Encapsulation efficiency of Cur was 87.77%; heat treatment and (85 °C, 120 min) UV irradiation (2 h): retention rate increased from 17.76% and 9.30% to 70.46% and 64.47%, respectively; in vitro antioxidant activity ↑	[9]
Rose essential oil (REO)	PPI–sodium alginate (NaAlg) complex coacervates	PPI/NaAlg ratio of 6:1, pH 3.8, REO/PPI-NaAlg = 1: 1.5; 10,000 rpm, 5 min	Encapsulation efficiency: 89.80%; payload: 53.17%; encapsulation yield: 88.26%; thermal stability and sustained-release profile ↑	[135]

“↑” stands for improvement.

## 5. Patents of Perilla Seed Processing

The bioactive compounds present in perilla are connected to several advantageous qualities, including anti-bacterial, anti-inflammatory, and antioxidant effects. Due to this, perilla is regarded as an advantageous asset in both contemporary pharmaceuticals and traditional medicine. The increasing number of international patents, mainly from Asia, with South Korea and China at the forefront, further illustrates the widespread interest in perilla. The inventive and varied uses of perilla in many industrial sectors are demonstrated by these patents.

The patents include a broad range of products with multiple uses, including novel formulations and delivery methods, as well as chemicals derived from perillas that have uses beyond conventional ones [137,138]. Patents on perilla seeds mainly focus on the processing and utilization of the oil. Products produced from perilla seed powder, for instance, have been developed to make use of the plant’s nutritional and health advantages while providing consumers with a stable and handy form [139]. Additionally, patents describing methods for the extraction, purification, and enhancement of oil quality suggest a significant focus on the industrialization of perilla oil production [140,141,142].

The patent landscape suggests a growing awareness of perilla’s potential in the global market, with researchers and industries alike investigating its diverse applications. This trend is likely to continue as more studies elucidate the intricate chemistry of perilla and its potential applications in health, nutrition, and other industries. The integration of traditional knowledge with modern scientific research and technology is poised to expand the horizons of perilla utilization, offering new opportunities for innovation and commercialization on a global scale.

## 6. Conclusions and Perspective

In recent years, plant-based diets have garnered widespread attention. Perilla seed is extensively utilized in the food and pharmaceutical industries due to its high contents of ω-3 polyunsaturated fatty acids and protein. This review focuses on the composition, health benefits, and application of perilla seed oil and protein. While significant progress has been made in research on perilla seed oil and protein, there are still a number of opportunities and challenges that need to be addressed.

Firstly, the high content of polyunsaturated fatty acids makes PSO susceptible to oxidation and requires the development of effective antioxidant strategies and preservation methods. Modern processing techniques such as supercritical CO_2_ extrusion, microencapsulation, and atomized drying can be used as effective means to treat and stabilize PSO. Secondly, the health benefits of PSO are currently limited to theoretical research based on in vitro and animal experiments, lacking an in-depth exploration of the underlying mechanisms of action. Therefore, the focus in the future should be on large-scale, long-term clinical trials to confirm the effects of PSO on human health and the molecular mechanisms of action and to determine appropriate dosage recommendations. Thirdly, researchers have focused most of their attention on studying the functional properties of perilla seed protein, while further exploration and development of efficient extraction techniques for perilla seed protein have not been pursued. Fourthly, compared to legume proteins, perilla seed protein has not been commercially applied at present. More research is needed to comprehensively understand the functional properties of perilla seed protein in various food systems and to utilize greener and more cost-effective protein modification techniques in order to increase the range of applications for perilla seed protein. Finally, exploring the synergistic effects of PSO or perilla seed protein with other functional ingredients could lead to the development of novel nutraceuticals or functional foods with enhanced health benefits.

In conclusion, perilla seed oil and protein represent valuable resources with significant potential in the functional food and nutraceutical industries. Continued research and development in this field will likely unveil new applications and benefits, further establishing perilla seeds as an important functional food source in the future.

## Data Availability

All relevant data have been presented as an integral part of this manuscript.
